# Challenges to ensuring valid and useful waiting time monitoring – a qualitative study in Swedish specialist care

**DOI:** 10.1186/s12913-021-07021-y

**Published:** 2021-09-28

**Authors:** David Ebbevi, Henna Hasson, Knut Lönnroth, Hanna Augustsson

**Affiliations:** 1 Unit for implementation and evaluation, Center for Epidemiology and Community Medicine Stockholm Regional Council, Stockholm, Sweden; 2 Astrid Lindgren Children’s Hospital, Karolinska University Hospital, Stockholm, Sweden; 3 Medical Management Centre, Department of Learning, Informatics, Management and Ethics, Karolinska Institutet, Stockholm, Sweden; 4 Department of Global Public Health, Karolinska Institutet, Stockholm, Sweden

**Keywords:** Access, Waiting times, Measurement, Quality registry, Health policy, Health system quality and safety

## Abstract

**Background:**

Access to health care is an essential health policy issue. In several countries, waiting time guarantees mandate set time limits for assessment and treatment. High-quality waiting time data are necessary to evaluate and improve waiting times. This study’s aim was to investigate health care providers and administrative management professionals’ perceptions of validity and usefulness of waiting time reporting in specialist care.

**Methods:**

Semi-structured interviews (*n* = 28) were conducted with administrative management and care professionals (line managers and care providers) in specialized clinics in the Stockholm Region, Sweden. Clinic-specific data from the waiting time registry was used in the care provider interviews to assess face validity. Clinics were purposefully sampled for maximum variation in complexity of care, volume of production, geographical location, private or public ownership, and local waiting times. Thematic analysis was used.

**Results:**

The waiting time registry was perceived to have low validity and usefulness. Perceived validity and usefulness were interconnected, with mechanisms that reinforced the connection. Structural and cognitive barriers to validity included technical and procedural errors, errors caused by role division, misinterpretation of guidelines, diverging interpretations of nonregulated cases and extensive willful manipulation of data.

**Conclusions:**

We identify four misconceptions underpinning the current waiting time reporting system: passive dissemination of guidelines is sufficient as implemented, cognitive load of care providers to report waiting times is negligible, soft-law regulation and presentation of outcome data is sufficient to drive improvement, and self-reported data linked to incentives poses a low risk of data corruption. To counter low validity and usefulness, we propose the following for policy makers and administrative management when developing and implementing waiting time monitoring: communicate guidelines with instructions for operationalization, address barriers to implementation, ensure quality through monitoring of implementation and adherence to guidelines, develop IT ontology together with professionals, avoid parallel measurement infrastructures, ensure waiting times are presented to suit management needs, provide timely waiting time data, enable the study of single cases, minimize manual data entry, and perform spot-checks or external validity checks. Several of these strategies should be transferable to waiting time monitoring in other contexts.

**Supplementary Information:**

The online version contains supplementary material available at 10.1186/s12913-021-07021-y.

## Background

Sustaining equitable access to health care is acknowledged as an essential health policy issue [1], and waiting times have received attention in access assessments [2]. The presence of waiting lists may have some benefit for a society if waiting times are reasonable, as this reduces the risk of expensive hospital services being underutilized [3, 4], and patients that would have been treated unnecessarily [5] might avoid potential treatment risks. However, health systems worldwide are struggling with long waiting times that cannot be justified from an efficacy perspective [6]. This has been aggravated by the COVID-19 pandemic [7]. Long waiting times may threaten the legitimacy of publicly financed health care systems [8]. To tackle this, several countries in the Organisation for Economic Co-operation and Development have introduced soft-law regulations through waiting time guarantees that guarantee health care visits and treatments within a set time limit [9]. To this end, waiting time data are collected and used at least on two levels: 1) at the policy level for reimbursement/economic sanctions, to compare care providers, and to plan care expansion and 2) at the clinic level in production planning and to increase transparency toward patients. To achieve these objectives, waiting time data must be accurate.

Great variance in waiting time data can be seen between clinics for similar medical services [10]. In addition, considerable variation in waiting times has even been observed between professionals at the same clinic [11]. Some of these variations might reflect real differences in waiting times, while some might be explained by divergent reporting and data aggregation practices. Differences between professionals and clinics in reporting practices related to waiting time data can be problematic from a credibility perspective. Previous studies have evaluated sampling procedures and aggregation techniques for waiting times using statistical theory, showing how specific collection methods lead to theoretical biases [12, 13]. Any data valuable to health care need to be trustworthy and useful for stakeholders. However, previous studies have not explored suggestions for ensuring correct waiting time data from the perspective of stakeholders such as professionals and health care administrators. In order to ensure the high accuracy of waiting time data, it is necessary to examine registration and reporting practices. The aim of the study was to investigate health care providers’ and administrative management professional’s perceptions of the validity and usefulness of waiting time reporting in specialist care.

## Methods

### Setting

The study was conducted in Stockholm Region, the largest health care provider organization in Sweden. Swedish health care is largely publicly funded, with low equivalent patient fees for public and private care providers. Health care is structured according to a provider/purchaser-model (sometimes known as the client/contractor-model) in which the execution of care is separated from the administrative management of health care (e.g., funding and care needs analysis).

This study focused on specialist care, which is separate from primary care and refers to care provided by health care providers specialized in areas other than general. Specialist clinics in Sweden are single-specialty, for example urology, psychiatry, pediatrics, ophthalmology and otorhinolaryngology. In the present study specialist care concerns consultation room practices and in hospital practices. Specialist appointments are available through referral from general practitioner, as hospital care follow-up or as emergency care follow-up. Waiting for referral is seldom an issue, and access to general practitioners was generally available within 7 days at the time of the study. In addition, some specialist clinics accept patients without referral. When booking appointments, specific practitioner is usually allocated by the clinic based on availability, but the patient may decline or ask for a certain medical professional.

Equal access to health care is one of the building blocks of the health care system, and national legislation stipulates maximum waiting times for appointments and treatments [14]. In addition, regional health care providers can have local policies for even shorter waiting times. In the Stockholm Region, there is a target of maximum waiting time of 30 days for appointments (measured from decision to refer to a specialist to the first appointment), and 90 days for treatments in specialist care (measured from the decision to treat to the actual treatment). Patients with serious illnesses as triaged for visits earlier than the maximum target. Exceptions from maximum target can be made on medical grounds known as medically motivated waiting (e.g., when a complicating co-morbidity temporarily prohibits a planned elective treatment) or when patients actively choose to wait longer (e.g., if the patient declines a booking or a certain professional). In these cases, the waiting time episode is assigned a label known as an exception code. This means that there is no time limit in, or monitoring of, the waiting time.

The measurement of waiting times in the Stockholm Region consist of two reporting systems: *the waiting time registry*, and *the supply service database*. For the waiting time registry, data are mainly extracted automatically from electronic medical records. However, exception codes are entered manually. The data in the waiting time registry is aggregated on the clinic level through an online platform that enables report extraction for patients, care providers and other stakeholders. Any care provider can request a login to extract data concerning their own clinic. Data are presented as the percentage of patients for whom waiting times were within the target time limit. For some health care providers economic sanctions are administered when the waiting time guarantee is not met, based on results in the waiting time registry.

The other reporting system for waiting time measurement, the supply service database, contains clinics’ self-reported estimations of short-term future waiting times (e.g., a median four-week waiting time for cholecystectomy). Data are presented through an online platform for care providers and administrative management. The purpose of the supply service database is to provide estimations of future waiting times to direct patients to an appropriate specialist care provider, not (as with the waiting time registry) to audit compliance. The information is used to refer patients to care units with shorter queues.

### Design

A qualitative interview study was conducted with administrative health care management and care professionals. Clinic-specific data from the waiting time registry were used in care provider interviews to capture waiting time face validity and enable further reflection. The design was chosen because explanations for variations in quantitative data are unexplored, and the registry data itself was not expected to explain the variation. There was no Patient and Public Involvement in the study. As a means of Public Engagement part of study conclusion were published in Sweden’s largest daily newspaper [15].

### Study participants and data collection

A total of 28 semi-structured interviews were performed between October 2017 and January 2018. These interviews included administrative management (*n* = 11) and care professionals (*n* = 17). Administrative management included persons with two functions: 1) purchasers having a contractual and auditing function, including assessing compliance with the waiting time guarantee, and 2) quality assurers working with the administration and development of the waiting time registry. Respondents from administrative management were sampled purposefully to represent different parts of the waiting time measurement and reporting process and different amounts of experience working with the system. Three respondents from administrative management declined to participate. They stated that they did so because they had nothing to say about the issue.

Included care providers consisted of clinic staff responsible for reporting to the waiting time registry and the supply service database (*n* = 8) and clinic line managers (*n* = 9). We used maximum variation sampling [16] to create diversity among included clinics in terms of complexity of care, volume of production, geographical location, ownership (i.e., private or public), degree of attainment of the waiting time guarantee (data from September 1, 2017, from the waiting time database, varying between 37 and 100%), and extent of recorded exception codes. A total of 29 clinics were contacted via the manager, and 20 declined. Reported reasons for declining were as follows: they had nothing to say about the issue (*n* = 1), the waiting time guarantee did not apply to their care process (n = 1), they did not have time (n = 1), or they did not reply (*n* = 14). A total of eight clinics participated and were represented by a clinic manager and a clinic staff member. One clinic participated with a clinic manager but no clinic staff member.

Sampling of respondents in the two groups continued until data saturation was achieved (with the exception of respondents’ accounts of manipulation, which was sparse in the data but difficult to recruit purposefully). Sampled respondents who did not reply via e-mail were invited by telephone. Administrative personnel were first invited by the clinical manager at the participating care unit. Written informed consent was given by all respondents. On the request of the respondents, three were interviewed as a group, and six were interviewed in groups of two (only participants of the same unit). The first and last author conducted the interviews. They had > 5 years of experience in conducting research interviews. Interviews ranged from 23 to 71 min each (mean = 43 min). The interviews took place at the respondent’s work to promote a familiar atmosphere.

A semi-structured interview guide was used to allow respondents to talk about unexpected issues [16]. The first draft of the interview guide was constructed based on the literature [2, 6, 8, 10, 11] and discussions with personnel working with administrative management (two of whom were later interviewed). Perceived usefulness was defined as the potential benefits of using the waiting time systems [17]. The interview guide was further revised iteratively when new concepts emerged as interviews were performed. The areas covered by the interview guide were chosen to illuminate aspects of validity: knowledge about and feasibility and usefulness of the waiting time guarantee, communication and information about the guarantee and registry, measurements and measurement properties, sources of error in reporting, and the structure of the results and reports. All areas were covered in all interviews, but the questions were tailored to the function of the respondents (Full interview guide is available in Additional file 1).

Because care providers rather seldom used the registry to export their own clinic data from the waiting time registry, they were in addition presented with their data and asked to reflect on how well the data represented their perceptions of the waiting time (i.e., face validity). One interview was not recorded for technical reasons, but it was captured in detailed notes. The rest were voice recorded, and field notes were taken.

### Analysis

The interviews were transcribed verbatim by an external transcriber and analyzed to capture manifest and latent content using thematic analysis [18]. The process is outlined with examples in Table 1. The first and last author performed familiarizing and coding of their respective interviews independently with inductive codes using Dedoose v.8.0 [19]. The first author performed the steps searching for themes, reviewing and defining (Table 1). Codes were grouped though comparing each code with each other code (i.e. constant comparison [20]),The themes were formed to maximize inner homogeneity and outer heterogeneity [16] but include overlap. When faced with ambiguities, interpretations were facilitated by field notes and discussed between the first and last author (i.e., investigator triangulation) [21]. For example, field notes were used when respondents gestured towards data points, and triangulation in analyzing if differences between public and private care were colored by respondent’s political conviction. Formal member checks were not used, but results were discussed with administrative management, two of whom were interviewed.

**Table 1:** Thematic analysis

Step	Description	Example from analysis in the present study
Familiarizing	Read data multiple times and note ideas	Ideas: • Confusion from several parallel systems • Difference between private and public ownership?
Coding	Apply open codes to data relevant to research question	Coding: • “to be able to see the gain in using the [the registry]. You need to be able to see the gain. One does only need to look to oneself, one does not do anything if one can’t see the gain.” coded as “see gain of registry”
Searching for themes	Group codes into initial subthemes and themes	Forming tentative subthemes (here with example of codes): • *Automatization* o Thought data was automatically extracted o The registry should be automatic o Don’t know the system but believe it’s mostly automatic • *Quality assurance of waiting time registry and reports* o Care provider in need of better support o Administering mistakes in registry o Field-visits would be valuable but have no time
Reviewing	Check if themes represent their codes and all relevant data	Merging subthemes and moving codes: • *Automatization* was merged into subtheme *technical errors*, and *Quality assurance of waiting time registry and reports* into *Low usefulness of system output* • Subtheme *Errors due to roles and responsibility* was moved from theme *The cognitive task of reporting* to *Structural barriers to validity* • Code “Two different registries” was moved from *Trust as a mechanism of perceived validity*, to *Technical errors*
Defining	Analyze for renaming themes and formulate explicit definitions	Renaming: • *The cognitive task of reporting* was renamed *Cognitive barriers to validity* • *Need for feedback and its impact on usefulness* was renamed *Self-reinforcing validity and usefulness*

*Notes*. Thematic analysis of the present study. The themes were created iteratively, moving back and forth between the outlined steps. Subthemes in italics. Themes were not mutually exclusive since the components of the system interacts. Eg. a particular problem at the cross-section of the IT-system and the working process could both be seen as a technical problem and a procedural problem

## Results

**Fig. 1:**
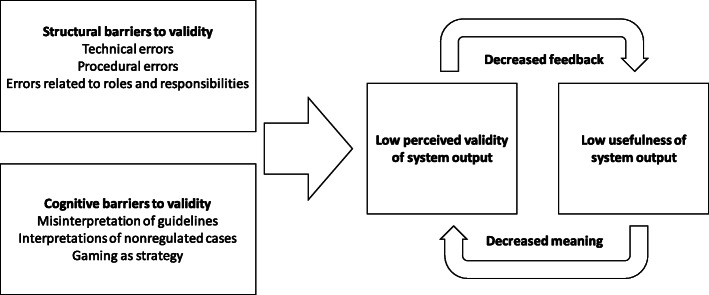
Interrelation of the themes. Subtheme self-reinforcing validity and usefulness is represented as decreased feedback & meaning

Respondents reported ambitions to shorten waiting times but found the two reporting systems ill-suited for that matter. Three themes describing the experience of waiting time data were found: interconnection between validity and usefulness, structural barriers to validity, and cognitive barriers to validity (Table 2). The relationships of the themes are illustrated in Fig. 1. Comparisons between the two stakeholder groups (care providers and administrative management) are made when differences are present in the data. Unless otherwise stated, the results refer to the waiting time registry, not the supply service database.

**Table 2:** Summary of findings

Themes	Subthemes	Defining the experience of...
**Interconnection of validity and usefulness**		how the value of a registry is determined by validity and usefulness
	Low usefulness of system output	usage of system output and suggestions for increased usefulness
	Low perceived validity of system output	perceived validity of waiting time data
	Self-reinforcing validity and usefulness	mechanisms for interaction of perceived validity and usefulness
**Structural barriers to validity**		errors in data caused by health care system structure
	Technical errors	errors in data caused by technical system, registry categories or technical system design.
	Procedural errors	errors in data caused by the structure of routines and care processes
	Errors due to roles and responsibilities	errors in data caused by role divisions between actors and self-perceived/explicit job descriptions.
**Cognitive barriers to validity**		errors in data caused by cognitive operations
	Misinterpretation of guidelines	error in data caused by misunderstanding or conscious misinterpretation of guidelines
	Interpretations of nonregulated cases	error in data caused by behaviors in situations not covered by the guidelines
	Manipulation as strategy	error in data caused by conscious sidestepping of guidelines to gain advantages

### Interconnection between validity and usefulness

The perceived value of the system was determined using the perceived validity and usefulness of the data output. This was salient among care providers and members of administrative management. Several respondents in administrative management thought incorrect data could still be useful as a starting point for discussion. Generally, the perceived usefulness and validity was low.

### Low usefulness of system output

The respondents raised several problems with the registry output. In addition to the slow and insufficient feedback on results (see the *self-reinforcing validity and usefulness* subtheme below), the measurements used in the registry were difficult to compare between time points. They suggested that reports with mean values for waiting times would be more useful in production planning and auditing compared to the current system reporting numbers and percentages of patients not receiving care within the set time limits.

Furthermore, purchasers felt exception codes had insufficient granularity. They also highlighted codes distinguishing medical conditions. For example, spinal care was coded together with other orthopedic care. This complicated their interpretation of the general orthopedic waiting times because spinal care often had a different waiting time level than general orthopedics did.

Care providers described four main approaches to using the waiting time data: 1) no use of the data, 2) individual patient spot-checks, 3) assessment of aggregated data, and 4) custom made data-collection and follow-up. Respondents making no use of the data (approach 1) said the data were outdated when they received it or that they did not trust the data. Thus, there was no reason for them to use the data at all. For them, these measurements and the data system provided no value. Respondents extracting data on individual level (approach 2) investigated the medical records to learn why each patient did not receive care within the guaranteed time. The waiting time registry was used to find patients who exceeded the waiting time guarantee, and specific electronic medical records were investigated for causes, either in a random sample of patients or in all the patients. Approach 3 concerned monitoring group level data for the clinic (approach 3) without investigating individuals. A care provider monitoring at group level explained as follows:

*There must be an opportunity for the clinics to control themselves to some extent. Every day, we have to adapt to political decisions that do not always go hand-in-hand with the medical needs we see, and we need to be able to adapt and do what’s best for the patient.*

Two care units had developed an additional system (approach 4) that collected data in parallel with the waiting time registry. The same data that were automatically reported to the waiting time registry were manually entered into a local database together with additional information that would enable further analysis, such as types of visit (elective vs. semi-elective). The care units could then analyze their local data to find improvements that would not have been attainable with data extracted via the waiting time registry.

### Low validity of system output

The trustworthiness of the data was perceived as low and often not aligned with the perception of waiting times. Large variations in validity were also perceived between care units, between medical appointments and treatments, and within patient groups. For example, the numbers could be perceived as correct for one type of surgery but incorrect for another at the same clinic, or correct at one time point but incorrect in another. The difference between treatment categories was especially problematic for a clinic responsible for surgery, orthopedics, gynecology, and urology at a larger hospital, because it gave treatments in about 30 different categories. The head of the clinic described the situation as follows:

*Yes, it is probably right for the prostheses because we are having trouble keeping up there. But most of the time, I think...God, yes... I do not think that’s right [pointing to the treatment category “Hand, Disease in Synovial Membrane”]. Yes, in that category, I think we have the resources. It has to be that we have failed [with the reporting]. It must be that for this kind of patient, we have been able to offer an earlier time.*

Several care providers described large errors:

*It’s one of five that is not operated on within three months [in your data]. It’s not concurrent with reality because we can offer a time next week to those who want it, and for those who do not want that time, the week after that. Every week, we have free spots, so maybe if someone says, “No, I want to be operated on in May,” then it will not be registered there.*

Nothing in the interviews indicated a connection between perceived validity and the respondents’ knowledge of the waiting time guarantee or the report system.

### Self-reinforcing validity and usefulness

Care providers requested quicker and more palpable feedback on their waiting times, either mediated by administrative management or by the registry report function. This emerged as the core mechanism by which validity and usefulness enforced each other. High validity would increase purchaser’s willingness to reach out to care providers about long waiting times, thus increasing registry usefulness. In addition, when care providers perceived the system and the data output as useful, they were more motivated to ensure high quality input data since it would otherwise lose its usefulness. When they made efforts to increase validity, they expected increased usefulness, and, conversely, if the system was perceived to have low usefulness, care providers lacked a sense of meaning and lost motivation to increase validity.

By contrast, some respondents from administrative management had a different view on validity. This was most evident when comparing the two reporting systems (i.e., the waiting time registry and the supply service database). Respondents working with the day-to-day maintenance of the waiting time registry, perceived the registry as more valid than the supply service database. They said that the data were more useful because they were not self-reported. However, among purchasers, some perceived the supply service database as more useful than the waiting time registry. Among those respondents, the supply service database was seen as having either higher or lower validity. They preferred the supply service database because the data were directly reported by care providers. It enabled a non-confrontational discussion with care providers about waiting time, compared to the waiting time registry that induced a feeling of auditing. In this case, the *actual* validity of waiting time data was not seen as a prerequisite to usefulness; perceived validity was sufficient.

Feedback was seen as a prerequisite to use the data when adjusting care production to meet waiting time targets. Some respondents did not want feedback from administrative management but wanted to monitor their own data. In terms of feedback timing, need differed from immediate access to access within a month of data registration. A manager at a care unit described it as follows:

*If you enter the visualization tool and try to be a little analytical and look at the numbers, then they are [at least two months old]. I tried to look at the referral flow now, because we have a feeling that it has increased, and feelings are rarely scientifically proven, and then it’s like numbers from three months back! I’m not helped by that. I want to see the number of referrals at least from the previous month, to be able to compensate...*

The two care units that developed an additional system (approach 4 in the *low usefulness of system output* subtheme) to collect data did so because the feedback from the registry was too slow and lacked sufficient detail, which limited understanding of reasons for not reaching the waiting time limits. A unit manager provided an example of the benefits of the additional system:

*We worked like crazy with the appointments, and yet the queue was longer than the year before. Then one asks, “Why is this so?”* And then we could see the answer in our system:*, “No, this is not due to elective visits, but emergency visits!” And those fluctuations are not within our control.*

The purchasers imagined that if the system is useful to care providers, they would feel more comfortable providing feedback to care providers. At the same time, many care providers and purchasers felt that the question of the timing of feedback loops was irrelevant because the data did not have high enough validity to be useful to them.

Some care providers wanted the administrative management to make a greater effort when enforcing contracts. Deviance from the waiting time guarantee was not sufficiently corrected (i.e., lack of feedback). Many purchasers agreed. This was described in statements about its necessity for system efficiency and could be understood as an issue of justice. A potential explanation for why the contracts were not enforced was suggested by a purchaser:

*It is not a big enough breach of contract, so you would make a lot of noise due to it, because you know that they want to attain to the waiting time guarantee. There is certainly no purpose for them not to do it.*

Care providers suggested that the steps taken to correct waiting times should be based on an analysis of waiting time causes rather than control through economic incentives.

### Structural barriers to validity

Respondents perceived that characteristics of the system, such as the technical design of the IT system, the procedural design of care delivery, and the distribution of responsibilities among the actors generated several errors in the output data.

### Technical errors

Technical errors consisted of errors due to parallel systems, automatic assignment, and incongruence between the care structure and registry terminology. This results in missing data in the first two cases and incorrect data presentation in the last case. First, some care providers had their own waiting lists in a digital structure that was not automatically synced to the waiting time registry. According to administrative management professionals, the personnel at the clinics were not aware that the list would not be synced to the waiting time registry, and were thus unaware that they were not reporting waiting times.

Second, since the system of transferring data from the electronic medical record to the waiting time registry was automatic, many providers assumed that the initiation of reporting was also automatic, when, in fact, the automatic reporting required a targeted setup before data transfer could occur automatically. Again, this means the care providers assumed that the data were synced to the registry, while this was not the case.

In addition, the electronic medical record supports several care professions, while the registry assumes that all visits concern medical doctors. This means that when reports were extracted from the registry, they portrayed waiting times for medical appointments incorrectly due to different access levels for other care categories. This was primarily a problem within specialties with a high number of visits compared to other care professions, such as rehabilitation that depends on physiotherapists and occupational therapists.

Finally, the decision to separate data into two data systems (the waiting time registry and the supply service database) was a cause for confusion, and respondents assumed they were connected. It was also common that care providers and purchasers could not distinguish between the two data systems although the two registries differed in terms of data sources, available variables, and user interface for data retrieval. A purchaser questioned the need for two parallel data sources:

*There are two different systems? Why? Do we really need two different data sources? Or should there only be one data source that’s “top notch,” and that’s the one that applies? Because it can be a little confusing to know which data I should look at.*

### Procedural errors

The care process was sometimes constructed in a way that required the sidestepping of procedures to avoid reporting errors to the registry. First, procedural errors occurred when patients changed care providers at a predetermined date (e.g., from pediatrics to adult care due to age). The referral time is counted as waiting. This means that if the referral is sent several months ahead of the care provider change, it appears in the system as though the patient has been waiting for too long. Second, a contract manager described how administratively different care processes can give rise to completely different data, even if the patient’s care is the same:

*If a child enters the children’s emergency ward and, from there, is referred to a follow-up appointment in the same hospital, it is recorded as a return visit. If the same child is referred from the emergency ward to an external elective follow-up, it will be considered a new visit.*

### Errors due to roles and responsibilities

Waiting time reporting was performed by different types of professionals at the various care providers. This created a risk of errors when individuals had to perform tasks beyond their perceived professional merit. For example, if a patient required rescheduling, the physician would typically forget to mark the rescheduling as patient-driven. Managers tried to keep physicians informed of the necessary process to avoid this error, but found it difficult:

*I cannot keep sitting in on each meeting saying, “Don’t forget to cancel a new visit via the secretary.” They have heard it! They do not internalize it, and remember, they are very competent. It’s just one of thousands of details, and we are exposed to even more orders and administrative details.*

A few units with an economic incentive to adhere to the waiting time guarantee used compensatory retrospective recording procedures to correct for this source of error. This was possible for the longer time limit of treatments (90d), but it was difficult for the shorter medical appointments (30d) because the professional would not find the time to do it before the time limit passed:

*It may often be that the secretaries enter this system and double-check. It takes a lot of time trying to prevent [error] and rebook, circumventing the system in some way. It is not like cheating, but they will have to create a fake booking because we have not done anything wrong.*

### Cognitive barriers to validity

Other errors were caused by the individuals involved in the reporting. Often, this was described as “forgetting.” This theme captures a sliding scale from misunderstandings through diverging interpretations to willful cheating.

### Misinterpretation of guidelines

The misunderstanding of guidelines in terms of how to enter data was salient. That is, respondents had an internalized understanding that was in direct conflict with the guidelines First, care providers made different interpretations of care flow, and this resulted in several errors. This means they used an incorrect starting point and endpoint for measurement. Both time points could be over- and underestimated. For example, as starting point in treatments, medical pre-examination was sometimes used (false short waiting time), while in other instances, the referral date was used as a starting point instead of the decision date (false long waiting time). A care provider described the lack of effort to correct this problem:

*Some count [waiting as the time] from the date [the patient was] referred to surgery, others count the time between preoperative assessment and surgery. And we have asked for clarity in terms of from how to count in Stockholm so all clinics can do it the same way, but it has not happened yet, though we have talked about this for several years.*

Another cause for confusion was medically motivated waiting. This guideline is intended for unexpected medically induced waiting. However, it was sometimes interpreted by care providers as any kind of medically motivated waiting. This could include appointments that were prioritized non-medically and thus could cover most patients with non-acute illnesses. However, the respondents did not use it consistently. In addition, medically motivated waiting was sometimes used if the patient needed to see the anesthesiologist first, needed an x-ray, or had to wait for a particular doctor because there was only one doctor with expertise in the area of the patient’s medical condition. While some respondents said they did this in good faith, others did it while aware that this was contrary to the guidelines:

*I think this is cheating, but you can code these [patients as waiting for] medical reasons, when there is a special doctor who has to handle the appointment. But we know that you should not use the codes that way.*

Other care providers instead categorized the exact same situation as patient-chosen waiting, as the care provider assumes that the patient wanted to wait for a doctor with expertise in their medical condition. In manifest data, this misinterpretation of the guidelines was a misunderstanding, but conscious misinterpretation could not be ruled out.

### Interpretations of nonregulated cases

The respondents also expressed instances in which the categories were not mutually exclusive or the guidelines were unclear. For example, in situations where a patient asks to wait on a treatment due to an acute event such as a fracture, this would fulfil the criteria for both patient-chosen waiting and medically-motivated waiting. The respondents found it difficult to make this distinction when reporting.

The reporters also found codes blunt and difficult to understand, and this made the selection of code categories unreliable. For example, in terms of termination codes, respondents lacked a category such as “Referral information not complete.”

### Manipulation as strategy

The respondents expressed several ways in which they thought others, or they themselves, adjusted reporting to suit the system. This was done irrespective of whether the unit had an economic incentive to report short waiting times. Some respondents sought to correct what they perceived as the unintentional side effects of the system. Some manipulation behavior could not be understood as correcting for system side effects. When care providers described manipulation among other care providers, they characterized it as cheating. A few care providers admitted to willfully manipulating the reports, while most said they were only aware that other care providers were doing it:

*There is a risk that you always register patient-chosen waiting, for example, if you cannot attain the waiting time guarantee. That could be a risk. We don’t do that, but if you are to compare with other clinics …*

Respondents also mentioned administratively postponing the final decision for treatment until right before treatment. That is, the patient would be put on an internal waiting list, then taken for medically unnecessary revisits, which are not covered by the waiting time guarantee. The revisits would then be used as time stamps for “treatment decisions,” so the time registered in the waiting time registry would be less than 90 days, even though the patients’ real waiting times in the cases described would be more than a year. In addition, manipulation of exception codes occurred, sometimes by offering a time no patient would want:

*We pretend that [...] patients have an appointment on New Year’s Eve. Nobody wants to come on New Year’s Eve. Then it will be “patient-chosen waiting” until mid-January. This is unpleasant; I think the system invites you to cheat.*

The knowledge of manipulation among care providers and purchasers also affected their perception of data validity. One care provider suggested spot-checks to correct for manipulation:

*I think it would be good to do a few more spot-checks on how care providers comply with these waiting time rules because I only hear that there are units and hospitals that cheat with the coding.*

By contrast, several respondents made an effort to comply with the system. They were not motivated by the usefulness or timeliness of reports, but by avoidance of economic sanctions and conformity to expectations.

## Discussion

The aim of this study was to investigate health care providers and administrative management’s perceptions of the validity and usefulness of waiting time reporting in specialist care. The main findings showed several challenges that influence the waiting time validity and, thus, the usefulness of the waiting time data. The main themes covered structural and cognitive barriers to validity and the interconnection of validity and usefulness. The current system is suggestive of four misconceptions: a) passive dissemination of guidelines is sufficient as implemented, b) the cognitive load of care providers to report waiting times is negligible, c) soft-law regulation and presentation of outcome data is sufficient to drive improvement, and d) self-reported data linked to incentives poses a low risk of data corruption. Individual respondents do not necessarily hold these misconceptions. On the contrary, representatives of administrative management highlighted care provider’s administrative burden (misconception b), and several of them suspected manipulation (d).

### Passive dissemination of guidelines is sufficient as implemented

Guidelines for reporting waiting times were communicated by administrative management in written form by e-mail. In most cases care providers did not adhere to the guidelines. Thus, the dissemination of guidelines was not sufficient for implementation. This is in line with implementation science, which shows that changing practices may require more comprehensive supporting activities than dissemination of guidelines [22]. Optimally, these activities should be tailored by local care providers based on potential barriers for implementation [22, 23]. The findings showed that barriers for correct reporting were not only lack of information or knowledge about how to report, but also care providers’ perceptions of the systems’ validity and usefulness as well as structural processes. Caregivers’ perceptions that the reporting system and the output data lacked validity and usefulness created a disincentive to report waiting times which, in turn, decreased the validity and usefulness of data. Processes used when moving patients between care units also impeded validity since any change in care unit was viewed as a new care process. This highlights the need for implementation support that would address these barriers.

Nevertheless, it is important for care providers to have some knowledge about the waiting time reporting system because the guidelines are not necessarily intuitive. For example, interpreting low clinical priority as medically motivated waiting was not according to guidelines, whereas that interpretation has been advocated in other contexts [24]. In addition, waiting time reporting categories and codes were not mutually exclusive, and were hard to make explicit in guidelines. Similar to our findings, other studies have noted that the granularity of the data in waiting time reports might cause biased interpretations [11], and certainly higher granularity of patients choosing to wait could enable analysis of equity of care. This emphasizes the importance of considering overlapping cases and deconstructing included categories when creating the IT ontology. Preferably, this could be done in collaboration with care providers. Distribution of guidelines without mechanisms to foster adherence might create a feeling of false security for guideline developers or administrative management [25]. The true waiting times might be hidden from view. This situation is especially unwieldy, as several actors have incentives to mask true waiting times: both care providers (to present short waiting times to administrative management), and administrative management (to present short waiting times to taxpayers and patients).

### Cognitive load of care providers to report waiting times is negligible

The waiting time system increased the cognitive load on care providers. For example, waiting times were issued in two parallel systems (the waiting time registry and the supply service database), based on separate data sets, variables, and target groups. In addition, the complicated assignment of exception codes increased cognitive load. As described by a respondent in *Errors due to roles and responsibilities*, correct reporting is not a matter of reminding care providers of instructions. Rather, there is too much to keep track of at the same time. Excessive demand on cognitive capacity can impair the uniformity of reporting and, hence, its quality. Parsimonious performance indicator systems have a greater chance of delivering high quality data than voluminous systems [26]. The number of waiting time indicators are few, but together with other performance indicators, the cognitive load from indicators is substantial. Therefore, a streamlined system usable to stakeholders could increase data quality. Earlier research on uptake and the validity of registry data highlights this aspect of usefulness (i.e., the relevance of the health care registries in daily work) [27]. For example, visual analytics in decision support systems can help health care personal with cognitive offloading [28].

### Soft-law regulation and presentation of data are sufficient to drive improvement

Many care providers were interested in their waiting times. For instance, the waiting time data were useful when referring patients to other care providers (in line with general practitioners in Great Britain [29]). However, this interest and intrinsic motivation was not sufficient to achieve waiting time improvements. The waiting time data were used to verify the current waiting times, but it is unclear whether it fueled behavior change and improvements. Care providers suggested that steps taken to correct waiting times should be based on an analysis of causes of long waiting times rather than regulated though economic sanctions. This aligns with earlier studies highlighting that data presentation is not enough to shorten queues [25].

Furthermore, respondents wished for more timely data presentation. Prior studies have showed that old data have little value and that continuous, real-time surveillance of waiting can be used for improvement [30]. This further aligns with a case description of queue management through data feedback in which real-time data and system usefulness enabled automated quality surveillance to achieve high data quality [31]. However, choosing time points for measurement of waiting times is complicated. For instance, although current waiting times (i.e., people waiting today) might seem more attractive than retrospective data in terms of timeliness, cross-sectional waiting times tend to underestimate the proportion of people with short waiting times when dropout is low and overestimate it if dropout is high, as compared to retrospective “true” waiting times [12].

### Self-reported data linked to incentives has a low risk of data corruption

The study found several potential errors in reporting, including manipulation. The data showed willful manipulation as well as slight adjustments under the pretense that the adjustment was morally justified. Manipulation is a problem in all performance measurement systems. Empirical studies show that waiting lists themselves [32, 33] and exception codes [34] are open to manipulation. Some show that complying with data collection may primarily aim to meet expectations rather than improve services [35], especially if presentation of data is combined with an incentive system [25]. Furthermore, care providers have been seen to game waiting list reporting even though it had economic disadvantages (i.e., they would have been given increased funding if the true length of the queues were known) [34]. This aligns with neo-institutional theory, which asserts that organizations are more interested in confirming certain expectations or values than increasing their own effectiveness [36, 37]. In the present study, data manipulation might have arisen from care providers seeking to give themselves economic, social, or regulatory advantages. Manipulation seems to be a behavior on the care unit level rather than the individual level; in other words, manipulation was often sanctioned by the head of the unit, as seen elsewhere [34, 38]. Reasonably, privately held care providers might be inclined to underrepresent the queue to avoid economic sanctions or ensure production at a maximum capacity (enhanced by a fee-for-service model), while publicly held clinics might be inclined to exaggerate the queue as economic loss is not carried by the clinics themselves, but by society. This view was prevalent among the respondents, but in practice, the private/public distinction was not a useful category for understanding the behavior of respondents. Rather, social mechanisms might be at play. The occurrence of a queue might signal the importance of the clinic to patients and referrers, and that increased funding might not mitigate the queue [39]. This aligns with the health economic assertion that medical interest is part of the physician’s utility function. That is, having a queue enables physicians to select the more interesting cases [40]. Finally, establishing a queue might enable care providers to motivate decreased regulation (e.g., alleviation of production limits).

Audit processes might mitigate manipulation, as perceived by the respondents. However, representatives of administrative management believed they lacked the resources necessary to investigate manipulation. This may constrain auditing even where manipulation is evident [34]. Making valid data useful to care providers is an important complimentary approach and should be built into technical infrastructure. Ideally a support system similar to those used for clinical quality registries could ensure that the data is comprehensive, correct and consistent.

### Suggested system-level strategies for waiting time measurements and reporting

We propose the following concrete strategies for administrative management of health care and policymakers to address the challenges from the unfounded assumptions discussed above (assumption ID in parentheses):

- Articulate and communicate guidelines with clear definitions and instructions for operationalization (a)
- Address barriers to implementation, monitor implementation and adherence to the guidelines, and alter the strategy if necessary (a)
- Develop the IT ontology together with care providers to ensure ease of use (a)
- Avoid parallel measurement infrastructures and integrate existing parallel reporting systems (b)
- Ensure that waiting time data are analyzed and presented in a way that corresponds to the actual planning and management needs of care providers and administrative management, not just to demonstrate whether targets are met (b)
- Provide timely waiting time data with feedback loops corresponding to planning cycles (c)
- Enable the study of single patient cases in waiting time statistics (e.g., by relating outliers to electronic medical registry) (c)
- Minimize manual data entry by optimizing functions for automated data extraction from electronic medical records (d)
- Perform spot-checks or external validity checks to enable quality improvement and to counteract manipulation (d)

### Methodological considerations

The qualitative approach used in one geographical area in Sweden limits generalizability. Key characteristics of the Swedish health care system, such as the publicly funded client-contractor model with patient choice reforms, affects the broader incentive structure and may limit the generalizability of the findings to other health care systems. For instance, the strategies suggested in the discussion were contextualized to this specific setting to increase operationalization. Transferring the strategies to other contexts is likely to require adaptation. The four unfounded assumptions are more transferable and can be used as a starting point in other contexts. In addition, key characteristics of the infrastructure and waiting time guarantee affect transferability. For example, several other countries already aggregate median waiting times [2], unlike Sweden, whereas other aspects (e.g., usage of similar exception codes [24]) closely mirror other high income health care systems.

Moreover, the low participation rate among the invited clinics is a concern for the coverage of potential respondents’ perspectives. The study was also performed during a limited period. A longitudinal study would have captured challenges and mechanisms as the case develops. This would have additionally illuminated the aim, as the study concerns a politically and technologically evolving area. Qualitative studies do not allow quantification of errors. Rather, the study explores potential errors and investigates potential mechanisms for those errors.

The ambition to study the system mandated the recruitment of respondents on multiple levels. This led to a sample of diverse subgroups in which trade-offs had to be made regarding depth and universality. The study attempted to capture sources of error and mechanisms across all specialties. Even though there were subgroup differences that could be explained in terms of specialty, saturation was not striven for within specialties. Often, only one person of a given specialty participated. To illuminate challenges in a specific subgroup such as waiting time errors in surgery, a separate targeted study would be necessary.

There is a risk that persons at the administrative management acted as gatekeepers and limited access to respondents of interest [41], as they gave suggestions on care providers of interest to include in the study. To mitigate this risk, the sample was expanded, and the identities of the individual care providers were not discussed with or disclosed to the administrative management.

The study was initiated and funded for by a unit at the Stockholm Regional Council, responsible for waiting time follow-up. Therefore, there is a risk that care providers were reluctant to provide truthful information concerning problems with their waiting time reports. For example, they might not have wanted to show lack of knowledge or interest around the waiting time systems. However, the fact that respondents provided rich information, positive and negative examples, including examples of manipulation, might suggest that this was not the case. To decrease this risk, respondents were carefully informed that results would only be reported in a way that they as individuals would not be possible to identify.

## Conclusion

The validity and usefulness of waiting time data are highly interconnected and reinforce each other, and the reporting of waiting times is challenged by structural and cognitive barriers. We identified concrete operational challenges with implementing guidelines for waiting time registration, limited perceived usefulness of the current data output, questionable effects of soft-law regulation, and the risk of manipulation in self-reported data. Careful consideration of these challenges is necessary in order for waiting time monitoring to fulfill its intended purpose and be a meaningful accountability system for tax-payers, policymakers, administrators, care providers, and patients.

## Supplementary Information

**Additional file 1.**

## Data Availability

The datasets generated and/or analysed during the current study are not publicly available due to integrity of participants but are available from the corresponding author on reasonable request.

## References

[1] Howden-Chapman P, Siri J, Chisholm E, Chapman R, Doll CNH, Capon A. SDG 3: ensure healthy lives and promote wellbeing for all at all ages. A guide to SDG interactions: from science to implementation. Paris: International Council for Science; 2017. p. 81–124.

[2] Viberg N, Forsberg BC, Borowitz M, Molin R (2013). International comparisons of waiting times in health care–limitations and prospects. Health Policy.

[3] Cooper RB. Introduction to queueing theory. North Holland: In Proceedings of the ACM'81 conference; 1981. p. 119–22.

[4] Goddard JA, Malek M, Tavakoli M (1995). An economic model of the market for hospital treatment for non-urgent conditions. Health Econ.

[5] Marden PF, Williamson M, Newton A, Robertson DA (2007). Revalidation of a colonoscopy waiting list by general practitioners is cost effective and reduces unnecessary endoscopy commissioning. Gastrointest Endosc.

[6] Siciliani L, Hurst J (2005). Tackling excessive waiting times for elective surgery: a comparative analysis of policies in 12 OECD countries. Health policy.

[7] Uimonen M, Kuitunen I, Paloneva J, Launonen AP, Ponkilainen V, Mattila VM (2021). The impact of the COVID-19 pandemic on waiting times for elective surgery patients: a multicenter study. PLoS One.

[8] Winblad U, Vrangbæk K, Östergren K (2010). Do the waiting-time guarantees in the Scandinavian countries empower patients?. Int J Public Sect Manag.

[9] Luigi S, Michael B, Valerie M. OECD Health policy studies waiting time policies in the Health sector what works?: what works? OECD Publishing; 2013.

[10] Dimakou S, Dimakou O, Basso HS (2015). Waiting time distribution in public health care: empirics and theory. Heal Econ Rev.

[11] Cromwell D, Griffiths D (2002). Waiting time information services: what are the implications of waiting list behaviour for their design?. Aust Health Rev.

[12] Armstrong PW (2000). First steps in analysing NHS waiting times: avoiding the ‘stationary and closed population’fallacy. Stat Med.

[13] Armstrong PW (2009). What do we know? Limitations of the two methods most commonly used to estimate the length of the prospective wait. Health Serv Manag Res.

[14] Hanning M. Maximum waiting-time guarantee-a remedy to long waiting lists?: assessment of the Swedish waiting-time guarantee policy 1992–1996: Acta Universitatis Upsaliensis; 2005.

[15] Ebbevi D, Hasson H, Lönnroth K, Magnusson C, Muli I. Forskare:” Statistiken om väntetider i specialistvården är opålitlig” - DN.SE [Internet]. [cited 2021 Aug 18]. Available from: https://www.dn.se/debatt/statistiken-om-vantetider-i-specialistvarden-ar-opalitlig/

[16] Patton MQ. Qualitative evaluation and research methods: SAGE Publications, inc; 1990.

[17] Davis FD (1989). Perceived usefulness, perceived ease of use, and user acceptance of information technology. MIS Q.

[18] Braun V, Clarke V (2006). Using thematic analysis in psychology. Qual Res Psychol.

[19] Version D (2017). Web application for managing, analyzing, and presenting qualitative and mixed method research data.

[20] Glaser BG, Strauss AL. Discovery of grounded theory: strategies for qualitative research: Routledge; 2017. 10.4324/9780203793206.

[21] Denzin NK. The research act: a theoretical introduction to sociological methods: Routledge; 1970.

[22] Baker R, Camosso-Stefinovic J, Gillies C, Shaw EJ, Cheater F, Flottorp S, et al. Tailored interventions to overcome identified barriers to change: effects on professional practice and health care outcomes. Cochrane Database Syst Rev. 2010;3(3):1–12. 10.1002/14651858.CD005470.pub2 PMC4164371 20238340

[23] Grol R, Grimshaw J (2003). From best evidence to best practice: effective implementation of change in patients’ care. Lancet.

[24] Godden S, Health AP-P, 2009 U. Waiting list and waiting time statistics in Britain : a critical review. publichealthjrnl.com. 2008;2007(July). 10.1016/j.puhe.2008.06.005 19054534

[25] Kenis P (2006). Waiting lists in Dutch health care: an analysis from an organization theoretical perspective. J Health Organ Manag.

[26] Carter N (1991). Learning to measure performance: The use of indicators in organizations. Public Adm.

[27] Essén A, Lindblad S (2013). Innovation as emergence in healthcare: unpacking change from within. Soc Sci Med.

[28] Simpao AF, Ahumada LM, Gálvez JA, Rehman MA (2014). A review of analytics and clinical informatics in health care. J Med Syst.

[29] Bennett J, Wadey L, Setters J (1988). Evaluation of a waiting list leaflet issued to general practitioners. J R Coll Gen Pract.

[30] Vezyridis P, Timmons S (2014). National targets, process transformation and local consequences in an NHS emergency department (ED): a qualitative study. BMC Emerg Med.

[31] Ju JC, Gan SA, JSW T, Huang PY, Mei CM, SMM W (2013). Managing patients’ wait time in specialist out-patient clinic using real-time data from existing queue management and ADT systems. Stud Health Technol Inform.

[32] Amoko DHA, Modrow RE, Tan JKH (1992). Surgical waiting lists I: definition, desired characteristics and uses. Healthcare management forum. Elsevier.

[33] Kreindler SA (2010). Policy strategies to reduce waits for elective care: a synthesis of international evidence. Br Med Bull.

[34] Buchanan DA, Storey J (2010). Don’t stop the clock: manipulating hospital waiting lists. J Health Organ Manag.

[35] McKevitt D, Lawton A (1996). The manager, the citizen, the politician and performance measures. Public Money & Management.

[36] Meyer JW, Rowan B (1977). Institutionalized organizations: formal structure as myth and ceremony. Am J Sociol.

[37] DiMaggio PJ, Powell WW (1991). The new institutionalism in organizational analysis.

[38] Lipley N (2009). Nursing staff warned not to’fiddle’waiting time figures: call for change in four-hour operational standard at RCN emergency care association conference. Emerg Nurse.

[39] Foote JL, North NH, Houston DJ (2004). Towards a systemic understanding of a hospital waiting list. J Health Organ Manag.

[40] Feldstein MS (1970). The rising price of physician’s services. Rev Econ Stat.

[41] Holloway I, Brown L, Shipway R (2010). Meaning not measurement: using ethnography to bring a deeper understanding to the participant experience of festivals and events. Int J Event Festiv Manag.

