# Supplementary material for: Challenges to ensuring valid and useful waiting time monitoring – a qualitative study in Swedish specialist care
Source: BMC Health Serv Res. 2021 Sep 28;21:1024. doi: 10.1186/s12913-021-07021-y (PMC8478272; doi:10.1186/s12913-021-07021-y)
Supplement: Supplementary file 1 — Additional file 1. [file 12913_2021_7021_MOESM1_ESM.docx]

# Additional file 1 - Interview guide

"Research questions" are in the back of the interviewers mind during the interview. They control which follow-up questions are asked, but are not asked themselves. The interview is based on 5 question areas (T1-T5) which are examined based on what the respondent has to say. Questions under each area are examples, and we explore what the respondent addresses (i.e. semi-structured interview). For example, if something unexpected and relevant comes up, we ask questions about this. Stakeholders refer to administration, care managers and employees in SLL.

# Research questions (thematic dimension)

A. How does the perception of the wait time guarantee and CVR differ between stakeholders? (T1)

B. How and by what means do stakeholders communicate information about the wait time guarantee? (T2)

C. What do stakeholders need for greater coherence of registry practices? (T2)

D. Do stakeholders perceive that measurements, functionality, infrastructure or implementation factors are lacking for follow-up of the wait time guarantee? (T2, T4, T5)

E. What sources of error can stakeholders identify themselves when reporting waiting times? (T4)

Q. To what extent do stakeholders perceive that the waiting time measurements represent what they intend to represent (i.e. face validity)? (T5)

G. Do organizational / structural conditions for reporting differ between care providers?

The question areas are the same for caregivers and compliance officers, but the sample questions differ since they have different perspectives.

## Example questions – Care unit managers and administrators

### Knowledge about and feasibility and usefulness of the waiting time guarantee (T1)

In what way do you notice the wait time guarantee in your working day?

Can you tell us what you know about the wait time guarantee?

Are there aspects of the wait time guarantee that you feel you do not understand?

How would you describe that you work with the wait time guarantee?

Do you see any conflict between the wait time guarantee and providing good care?

Would you need to know something about the wait time guarantee that you do not know today? What?

How do you perceive that the wait time guarantee affects the finances of your care unit?

### Communication about the guarantee and registry (T2)

How did you first get information about the wait time guarantee?

Do you feel that the information / instructions given about the wait time guarantee are clear and understandable? Who gives the instructions?

Do you know about the central waiting time register? What do you know about it?

Do you monitor the wait time guarantee yourself at the clinic?

Do you feel confident in how you / your business will use the electronic medical record for correct reporting? How did you learn about it?

Do you lack any information on how to report waiting times?

### Information to patients (T3)

How do patients get information about the wait time guarantee?

Which information channel do you think is most important for patients?

What information is given to patients about the wait time guarantee? In what contexts?

Do you think there is a connection between the information you give to patients and the wait times at your clinic?

Do you provide different information to different patients? How?

Do you usually take something special into account in patients when you / your business provides information about the wait time guarantee?

Can you tell us about an occasion when you needed to clarify or provide extra information about wait times?

Can you tell us about a common misunderstanding about the wait time guarantee? How do you usually handle it?

Do you sometimes do something to make sure that the patient has understood the information? If so, what? Why / why not?

Does it sometimes happen that the patient does not want the information? Why?

Do you use printed or electronic information material? Can you recommend any information material that we can look at to get an idea of ​​how patients are informed?

### Measurements, measurement properties and sources of error in reporting (T4)

Who decides what is reported?

Can you tell us in as much detail as possible how visits are coded for the wait time guarantee? Can you tell us in as much detail as possible how you report for the wait time guarantee? By who?

Do you think that what you report for the wait time guarantee is meaningful?

Is it common for patients to choose to wait? How do you find out? Can you give more examples?

What does medically justified waiting mean? Who decides if waiting is medically justified?

Has it ever happened that you reported in a certain way according to the wait time guarantee but then regretted it afterwards? What happened?

What sources of error do you experience occur in the reporting of waiting times?

### The structure of the results and reports (T5)

How is care affected by the reporting of the wait time guarantee?

Do you know how you are doing in terms of waiting times?

To what extent do you feel that the image provided by reported waiting time data is in line with your perception of wait times?

[Local waiting times are displayed to the respondent] Does this agree with how you perceive your waiting times?

## Example questions - HSF

### Knowledge about and feasibility and usefulness of the waiting time guarantee (T1)

Tell us about your relationship with the wait time guarantee?

In what way do you notice the wait time guarantee in your working day?

Are there aspects of the wait time guarantee that you feel you do not understand?

How would you describe the wait time guarantee for your function?

Do you see any conflict between the wait time guarantee and providing good care?

Would you need to know something about the wait time guarantee that you do not know today? What?

### Communication about the guarantee and registry (T2)

How did you get information about the wait time guarantee?

Do you feel that the instructions given about the wait time guarantee are clear and understandable? Who gives the instructions?

Tell us about your relationship with the central waiting time register?

How often and under what conditions do you follow up on the wait time guarantee?

Describe how the electronic medical records affects the correctness of reporting? How did you find out about the correctness?

Do you lack any information regarding the waiting time reporting to the register?

How do you think caregivers receive information about the wait time guarantee?

Can you tell us about a common misunderstanding of the wait time guarantee? How do you usually handle it?

Does it happen that caregivers are uninterested in the wait time guarantee? Why?

### Information for patients (T3)

How do you perceive patients receive information about the wait time guarantee?

Which information channels do you think are most important for patients?

What information is given to patients about the wait time guarantee? In what contexts?

### Measurements, measurement properties and sources of error in reporting (T4)

Who decides what is reported?

Can you tell us in as much detail as possible how you would like visits to be coded? By who?

Do you think that what is reported for the wait time guarantee is meaningful?

How do you perceive patient-chosen waiting or medically motivated waiting?

Would you like to add other measurements to the registry?

## The structure of the results and reports (T5)

How is care affected by the reporting of the wait time guarantee?

To what extent do you feel that reported waiting time data is in line with your perception of waiting times?

# Examples of follow-up questions / clarifications

What is the main reason for…?

Have you experienced any conflict between X and Y?

Have you ever…?

Do you think there is a connection between X and Y?

Can you tell me about…

Do you ever remember that…

What happened in this and this episode you mentioned?

Can you describe in as much detail as possible any situation when…?

How do you think your colleagues look at ..?

Can you say something more about…?

Can you give some more examples?

Can you describe it one more time in a little more detail?
